# Multifaceted roles of zinc finger proteins in regulating various agronomic traits in rice

**DOI:** 10.3389/fpls.2022.974396

**Published:** 2022-07-25

**Authors:** Yifeng Huang, Longgang Du, Meixi Wang, Mengyun Ren, Shouwu Yu, Qianying Yang

**Affiliations:** ^1^Institute of Crop and Nuclear Technology Utilization, Zhejiang Academy of Agricultural Science, Hangzhou, China; ^2^Guangdong Province Key Laboratory of Plant Molecular Breeding, Guangzhou, China; ^3^Division of Integrative Bioscience and Biotechnology, Department of Life Sciences, Pohang University of Science and Technology (POSTECH), Pohang-si, South Korea

**Keywords:** rice, transcription factor, zinc finger protein, yield, phytohormone, stress response

## Abstract

Rice is an important cereal crop, which provides staple food for more than half of the world's population. To meet the demand of the ever-growing population in the next few decades, an extra increase in rice yield is an urgent need. Given that various agronomic traits contribute to the yield of rice, deciphering the key regulators involved in multiple agronomic trait formation is particularly important. As a superfamily of transcription factors, zinc finger proteins participate in regulating multiple genes in almost every stage of rice growth and development. Therefore, understanding zinc finger proteins underlying regulatory network would provide insights into the regulation of agronomic traits in rice. To this end, we intend to summarize the current advances in zinc finger proteins, with emphasis on C2H2 and CCCH proteins, and then discuss their potential in improving rice yield.

## Introduction

Rice crops are prone to various types of stresses, including biotic and abiotic stresses (Atkinson et al., 2013). Several gene families are induced and encode functional proteins (metabolic proteins) that directly protect rice crops against stresses or encode regulatory proteins that regulate the signal transduction in response to stresses (Jan et al., 2013). The regulatory proteins include transcription factors, protein kinases, protein phosphatases, and proteins involved in inorganic phosphate turnover (Han et al., 2020).

Zinc finger proteins that contain the zinc finger domain constitute one of the largest transcription factor families in eukaryotes (Seetharam and Stuart, 2013). The zinc finger was defined as the motifs in which cysteines and/or histidines coordinate a zinc atom(s) to form local peptide structures (Takatsuji, 1998). Zinc finger proteins participate in transcriptional regulation, RNA binding, regulation of apoptosis, and protein–protein interactions and play important roles in growth, development, and responses to environmental stresses (Takatsuji, 1998; Agarwal et al., 2007; Ciftci-Yilmaz and Mittler, 2008; Han et al., 2020).

Zinc finger proteins have been classified into different categories by different researchers. One of the classifications includes nine subfamilies, namely, Cys2/His2-type (C2H2), Cys3His (C3H), Cys3HisCys4 (C3HC4), Cys2HisCys5 (C2HC5), Cys4HisCys3 (C4HC3), Cys2HisCys (C2HC), Cys4 (C4), Cys6 (C6), and Cys8 (C8), based on their conserved cysteine (Cys) and histidine (His) residues that fold into a finger-like structure (Cassandri et al., 2017). Another classification includes six groups, namely, C2H2, C3H, C2C2, A20/AN1, C3H2C3, and C3HC4, based on the sequence characteristic of zinc finger conserved domain (Jin et al., 2018). Zinc finger is also classified into six groups, namely, C2H2, C2C2, C2HC, C2C2C2C2, C2HCC2C2, and CCCH, based on the number and order of the Cys and His residues that bind the zinc ion (Ciftci-Yilmaz and Mittler, 2008; Jan et al., 2013). The zinc domain types include C2H2, C8, C6, C3HC4, C2HC, C2HC5, C4, C3H, and C4HC3 (Berg and Shi, 1996).

Among different types of zinc finger proteins, the C2H2 zinc finger proteins constitute one of the largest families of transcriptional regulators in plants, with 176 members in *Arabidopsis thaliana* and 189 members in rice (Agarwal et al., 2007; Ciftci-Yilmaz and Mittler, 2008). The C2H2 proteins contain a conserved QALGGH sequence within their zinc finger domain (Takatsuji, 1998). The CCCH zinc finger proteins have been known to play important roles as RNA-binding proteins in animals (Wang, D. et al., 2008). A total of 67 and 68 CCCH zinc finger protein genes were found in rice and Arabidopsis, respectively (Wang, D. et al., 2008). There are 12 members of A20/AN1-type zinc finger proteins in rice, and 10 members in Arabidopsis (Huang et al., 2008). The classification of zinc finger protein genes can be different based on different family assignment rules (http://planttfdb.gao-lab.org/help_famschema.php). For example, *japonica* rice contains 135 C2H2 genes and 74 C3H genes according to the PlantTFDB v5.0 database (Tian et al., 2020).

**Figure 1:**
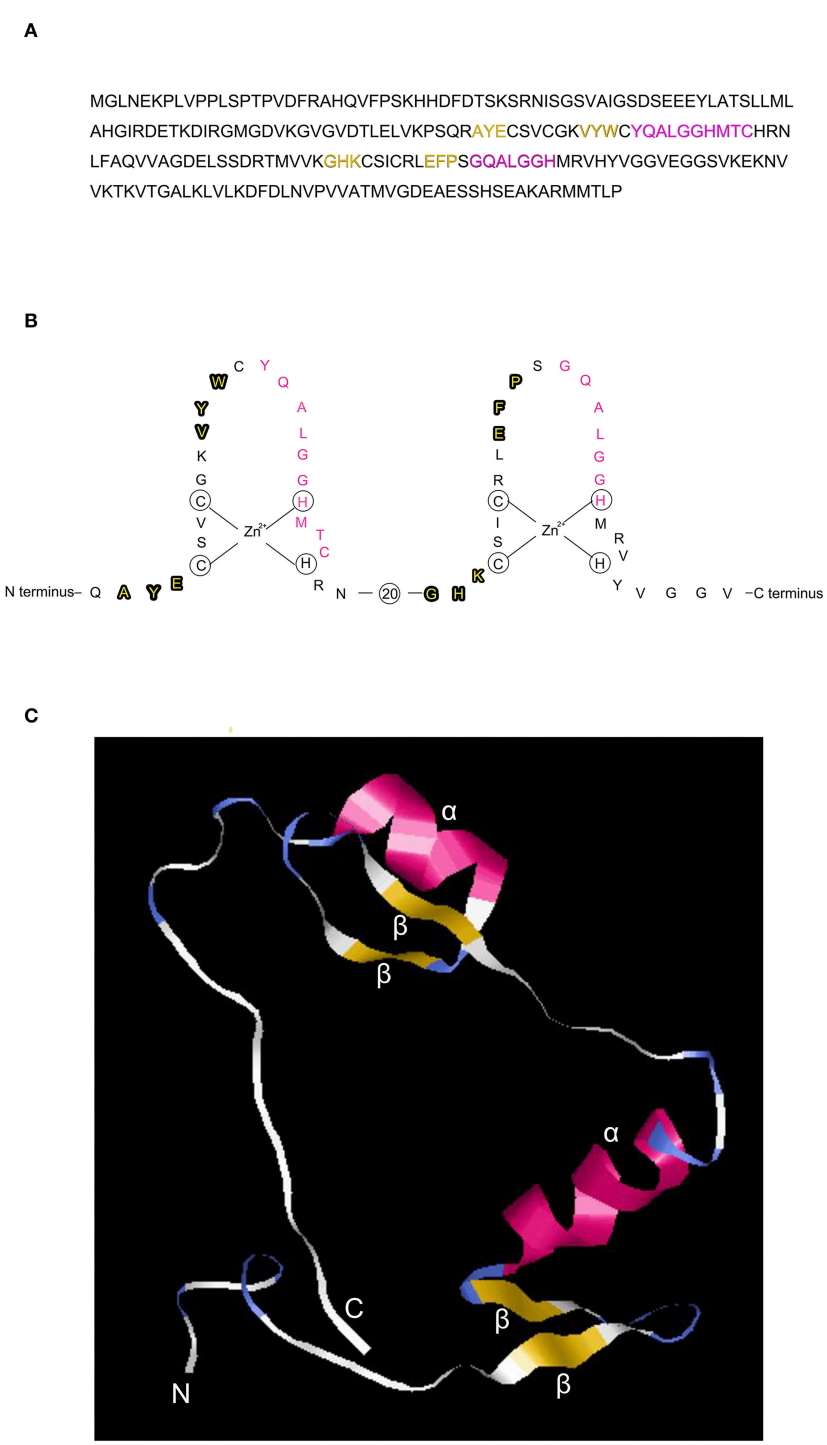
Structure of the C2H2-type zinc finger transcription factor ZFP39. **(A)** Amino acid sequence of the zinc finger protein ZFP39 (GenBank: AAQ95583.1) containing two tandem fingers, each finger has two β strands (in yellow) and one α helix (in pink). **(B)** Structure of the two tandem zinc finger motifs, 20 indicates 20 amino acid residues omitted between the two fingers for conciseness. The β strand and α helix of the zinc finger protein are highlighted in bold and pink, respectively. **(C)** 3D structure of the two tandem zinc fingers demonstrated using phyre2 (Protein Homology/analogY Recognition Engine V 2.0) (http://www.sbg.bio.ic.ac.uk/phyre2/html/page.cgi?id=index) and shown by the RasMol software (http://www.rasmol.org). N and C indicate the N terminal and C terminal, respectively. β, β strand. α, α helix.

**Figure 2:**
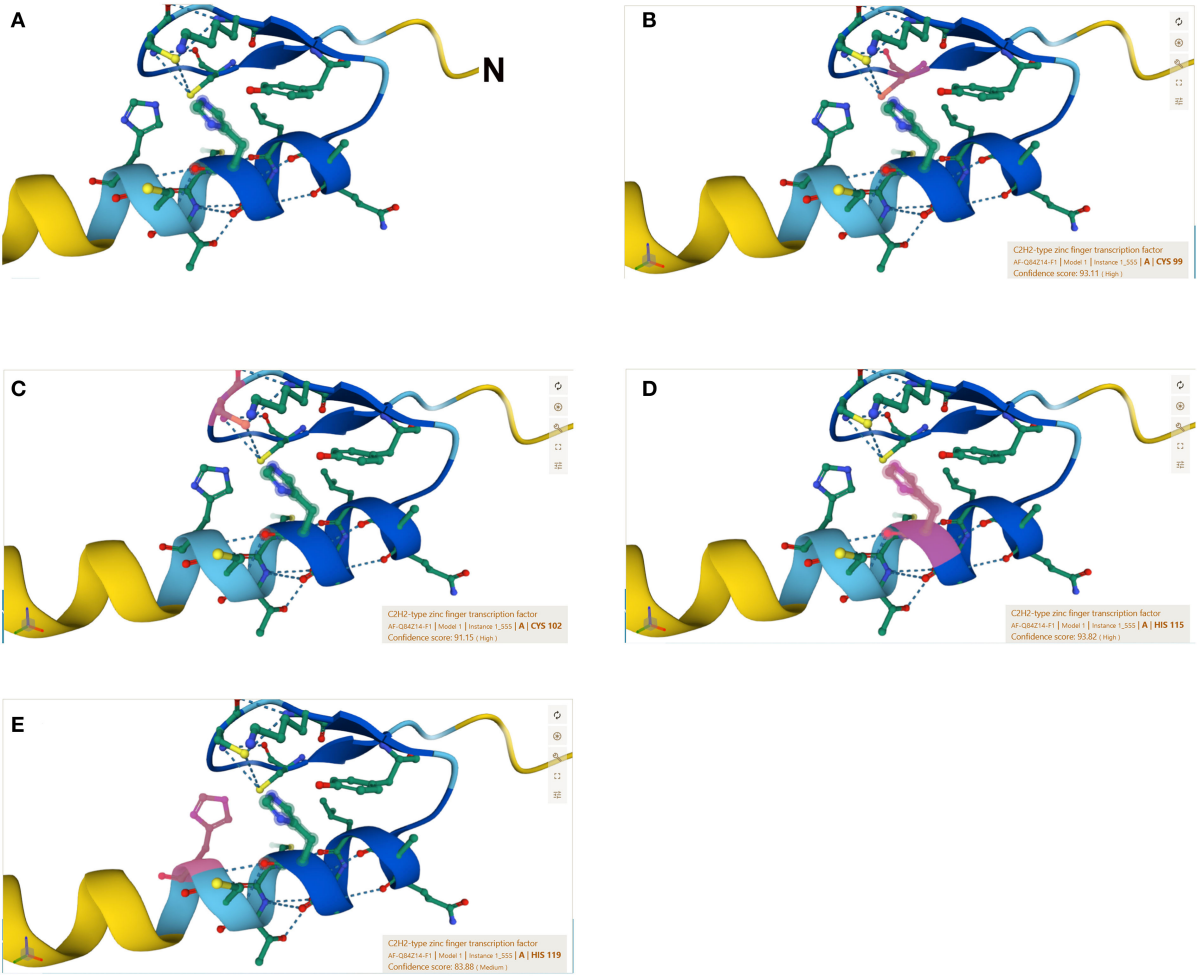
The β*βα* amino acid sequence of the ZFP39. **(A)** Particle ZFP39 sequence forming the β*βα* structure: N terminal-PSQRAYECSVCGKVYWCYQALGGHMTCHRNLFAQ-C terminal. See Figure 1A for the full amino acid sequence. **(B)** Pink color showing the 99th amino acid cysteine of the ZFP39. **(C)** Pink color showing the 102th amino acid cysteine of the ZFP39. **(D)** Pink color showing the 115th amino acid histidine of the ZFP39. **(E)** Pink color showing the 119th amino acid histidine of the ZFP39. The zinc is tetrahedrally coordinated between two cysteines at the β-sheet and two histidines at the α-helix.

The C2H2 zinc finger has a consensus sequence CX2-4CX3FX5LX2HX3-5H, where X represents any amino acid (Huang et al., 2012). The 3D structure of the zinc finger domain has been determined using Xenopus protein Xfin-31, which contains a YXCX2CX3FX5LX2HX3H sequence where the highly conserved residues are underlined (Lee et al., 1989). Most plant C2H2 zinc finger proteins have the highly conserved sequence QALGGH (Han et al., 2020). In this study, we use a rice C2H2 zinc finger transcription factor protein (GenBank: AAQ95583.1) to illustrate the zinc finger structure that contains two tandem finger motifs (Figures 1A,B), each motif having two b strands and one a-helix (Figure 1C) and (Figure 2A). The two b strands are arranged in a hairpin structure, accompanied by the a-helix and form a compact b b a domain in the presence of zinc (Wolfe et al., 2000). The zinc is tetrahedrally coordinated between two cysteines at the b-sheet (Figures 2B,C) and two histidines at the a-helix (Figures 2D,E).

## From DNA recognition to biological function

The unfavorable environmental conditions will induce successive processes, including signal perception, signal transduction, transcription regulation, and stress-responsive gene expression (Baillo et al., 2019). In the process of transcription regulation, transcription factors bind with DNA elements and recruit additional proteins. Some transcription factors access and open the closed chromatin to permit other transcription factors bind with the specific DNA (Strader et al., 2022). The zinc finger protein Zif268, which contains three zinc fingers, binds the major groove of B-DNA and wraps the partway around the double helix (Pavletich and Pabo, 1991). DNA recognition usually requires two to four tandemly arranged zinc fingers and additional secondary structure elements (Wolfe et al., 2000). Studies have shown that an additional 20-residue proximal accessory region (PAR), at the N-terminal to the first zinc finger domain, is required for high-affinity DNA binding with C2H2 zinc finger protein ADR1, and the mutations in the PAR lead to a loss of high-affinity DNA binding (Bowers et al., 1999). When plants suffer from abiotic or biotic stress, the plant zinc finger proteins bind to the *cis*-elements of stress-related genes by DNA contact and recognition, subsequently recruiting additional proteins to enhance or suppress the transcription levels of stress-related genes, thus regulating the cell division, growth, and development.

## Interplay with phytohormones to regulate agronomic traits

In this review, we mainly focus on the research advances in the involvement of zinc finger proteins in phytohormones-mediated agronomic traits in rice.

### Gibberellin

**Figure 3:**
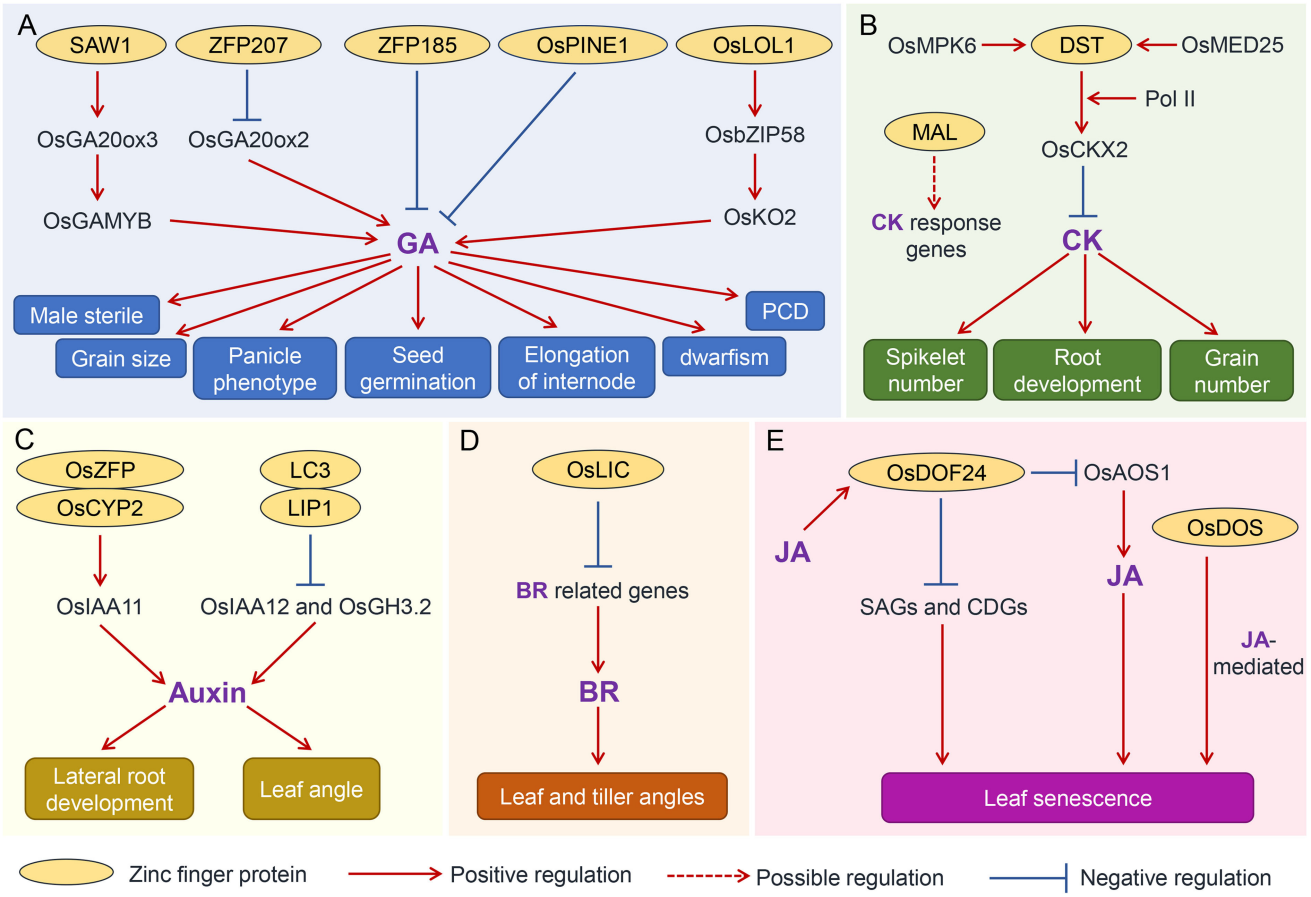
The proposed regulatory network of zinc finger proteins and phytohormones pathways in determining agronomic traits in rice. The regulatory network of zinc finger protein and GA **(A)**, CK **(B)**, auxin **(C)**, BR **(D)**, and JA **(E)**. Positive and negative regulations of various agronomic traits mediated by different zinc finger proteins are indicated by solid lines, and the proposed regulation of zinc finger proteins on downstream targets is indicated by the dashed line. The proteins with cycle are the zinc finger proteins. GA, gibberellins; CK, cytokinin; BR, brassinosteroid; JA, jasmonic acid. SAW1, Swollen Anther Wall 1; OsGA20ox3, GA biosynthetic gene; OsGAMYB, anther-regulator gene; OsGA20ox2, GA biosynthetic gene; OSPINE1, premature internode elongation 1; OSKO2, GA biosynthetic gene; DST, drought and salt tolerance; OsMED25, mediator subunit; OsCKX2, cytokinin oxidase 2; MAL, meristem activityless; LC3, leaf inclination 3; LIP1, LC3-interacting protein 1; OsLIC, tiller angle increased controller; OsDOF24, DNA-binding one zinc finger 24; CDGs chlorophyll degradation genes; SAGs, senescence-associated genes; OsAOS1, JA biosynthetic gene.

Gibberellin (GA) is an important phytohormone governing several agronomic traits (Figure 3A). Knockout of the *Swollen Anther Wall 1* (*SAW1*), encoding a CCCH-tandem zinc finger protein, resulted in male sterility. SAW1 directly targets the promoter of the GA biosynthetic gene *OsGA20ox3*, which triggers the synthesis of bioactive GA that is required for the induction of the anther-regulator gene *OsGAMYB*. The SAW1-OsGA20ox3-OsGAMYB module promotes the anther development (Wang et al., 2020). In contrast, another zinc finger protein ZFP207 (Cys2/His2 type) negatively regulates the GA synthesis by binding to the *OsGA20ox2* (also termed as Green Revolution Gene *SD1*) promoter to repress its expression, which leads to dwarfism, short grain and panicle phenotypes in rice (Duan et al., 2020). Overexpression of *ZFP207* caused a reduction in pollen viability (Duan et al., 2020). Another research showed that OsLOL1 (C2C2-type zinc finger protein) interacted with OsbZIP58 to regulate GA content by *OsbZIP58*-mediated activation of another GA biosynthetic gene *OsKO2*, which promotes the programmed cell death (PCD) and seed germination in rice (Wu et al., 2014). ZFP185 (A20/AN1-type) plays a distinct role in GA biosynthesis. Overexpression of ZFP185 in rice resulted in a decrease in endogenous GA3 content and then led to a semi-dwarfism phenotype (Zhang, Y. et al., 2016). A recent study revealed that a rice zinc finger protein *PREMATURE INTERNODE ELONGATION 1* (*PINE1*) also negatively regulates GA before the reproductive switch, thereby restraining the elongation of the internode. Then, florigens repress *PINE1* to promote stem responsiveness to GA and flowering (Gómez-Ariza et al., 2019). Furthermore, it is revealed that this *PINE1* is identical to the *DECELERATOR OF INTERNODE ELONGATION 1* (*DEC1*), which regulates the internode elongation antagonistically with an unknown function gene *ACCELERATOR OF INTERNODE ELONGATION 1* (ACE1). *ACE1* and *DEC1* are highly conserved in the Gramineae family, rendering the plants environmental fitness (Nagai et al., 2020). Together, these findings elaborate the diverse roles of zinc finger proteins in the GA underlying relevant traits.

### Cytokinins

Cytokinin (CK) is a vital phytohormone that regulates the spikelet number and grain number of rice (Figure 3B). *Gn1a*/*OsCKX2* (*Grain number 1a*/*Cytokinin oxidase 2*) has been proved to contribute to the grain yield. A C2H2-type zinc finger transcription factor DROUGHT AND SALT TOLERANCE (DST) was recently implicated to directly bind the promoter of *OsCKX2* and then trigger its expression, consequently orchestrating CK level to increase grain number (Li et al., 2013). Subsequently, the OsMPK6 was found to interact with and phosphorylate DST to promote *OsCKX2* expression and thus modulate spikelet number per panicle (Guo et al., 2020). In addition, the mediator subunit OsMED25 was also shown to act as a coactivator of DST, facilitating the recruitment of the RNA polymerase II (Pol II) to activate *OsCKX2* transcription. This regulation regulates the level of CK, ultimately resulting in the change in spikelet number in rice (Lin et al., 2022). Considering the contribution of CK content to grain number, DST-OsCKX2 module provides a promising potential for improving rice yield by modifying different alleles combinations. In addition, a recent report also illustrated that the *MERISTEM ACTIVITYLESS* (*MAL*), encoding a RING-H2 finger domain (RFD)-containing protein, might also coordinate the CK signaling to modulate root development by regulating CK responsive genes (Jiang et al., 2020). However, the direct target of MAL is still unknown, which would be an quite interesting issue to be addressed in order to manipulate the root development for drought tolerance improvement.

### Auxin

Auxin participates in the regulation of rice yield by regulating the grain yield too (Cao et al., 2020). However, the crosstalk between zinc finger proteins and auxin underlying this trait is unclear. But their relationships in regulating other traits have been explored (Figure 3C). For example, a C2HC-type zinc finger protein OsZFP interacted with the auxin-responsive gene OsCYP2 to modulate the lateral root development by orchestrating the expression of auxin signaling components, such as *OsIAA11* (Cui et al., 2017). Leaf inclination regulation is one of the most significant features of auxin in regulating agronomic traits (Li et al., 2020). The leaf inclination3 (LC3)-interacting protein 1 (LIP1), a HIT zinc finger domain-containing protein, directly binds to the promoter regions of *OsIAA12* and *OsGH3.2* and then suppresses the auxin signaling, resulting in the change of leaf angle (Chen et al., 2018). The regulation of zinc finger proteins coordinating auxin to modulate yield and quality-related agronomic traits is still elusive and deserved to be uncovered in the future.

### Brassinosteroids

Brassinosteroids (BRs) are the dominant phytohormone responsible for the regulation of leaf angle (Figure 3D). The *Oraza sativa leaf and tiller angle increased controller* (*OsLIC*) encodes a CCCH-type zinc finger protein, participating in modulating leaf and tiller angles. Microarray profiling revealed that expressions of BRs-related genes were significantly activated in the knockdown lines. Furthermore, BR treatment induced the expression of *OsLIC*, and sterol levels were higher in *oslic* mutants (Wang, L. et al., 2008), indicating that it might integrate the BRs pathway to determine the leaf angle. Considering *OsLIC* is a transcription activator, it coordinates with another transcription factor to directly suppress BR biosynthesis and/or signaling regulator expressions to orchestrate the BR response.

### Jasmonic acid

The phytohormone jasmonic acid (JA) modulates the timing of leaf senescence, which is an important agronomic trait contributing to rice yield, in particular during grain filling (Figure 3E). Recently, a rice *DNA-Binding One Zinc Finger 24* (*OsDOF24*) has been implicated to negatively regulate leaf senescence in rice by repressing the expression of senescence-associated genes (*Osl85, Osl57*, and *OsNAP*) and chlorophyll degradation genes (*NYC1, NYC3*, and *SGR*). Further analysis revealed that *OsDOF24* is specifically induced by methyl jasmonate (JA), and then, in turn, it inhibits JA biosynthesis by directly binding the promoter of JA biosynthetic gene *OsAOS1* and then probably represses its expression to modulate leaf senescence (Shim et al., 2019). In addition, anther zinc finger genes, *OsDOS*, has also been reported to be involved in the JA-mediated leaf senescence (Kong et al., 2006). For example, overexpression of *OsDOS* produced a delay of leaf senescence, while knockdown of *OsDOS* caused an accelerated leaf senescence. Subsequently, microarray analyses indicated that there were a number of differentially expressed genes involved in the JA pathway, suggesting *OsDOS* coordinates with JA to regulate leaf senescence (Kong et al., 2006). In summary, the zinc finger genes reviewed in this study are listed (Table 1), and the relevant regulatory mechanisms underlying agronomic traits are proposed (Figure 3).

**Table 1:** Representatives of the zinc finger protein in rice.

**Gene name**	**Type**	**Locus**	**Chr**.	**Function in agronomic traits**	**References**
DST	C2H2	LOC_Os03g57240	3	Negatively regulating drought and salt tolerance, leaf width, panicle length, grain number and heading date	Huang et al., 2009
LRG1/NSG	C2H2	LOC_Os04g36650	4	Negatively regulating spikelet and grain length, and grain width	Xu et al., 2020; Zhuang et al., 2020
OsCTZFP8	C2H2	LOC_Os08g20580	8	Positively regulating cold tolerance, pollen fertility and seed setting under cold stress	Jin et al., 2018
OsDRZ1	C2H2	Os11g0340477 ^*^	11	Positively regulating drought resistance, plant height, panicle size, and grain weight.	Yuan et al., 2018
OsIDD2	C2H2	LOC_Os01g09850	1	Negatively regulating plant height and leaf strength	Huang, P. et al., 2018
OsMSR15	C2H2	LOC_Os03g41390	3	Positively regulating drought tolerance	Zhang, X. et al., 2016
OsZFP213	C2H2	LOC_Os12g42250	12	Positively regulating salt tolerance	Zhang, Z. et al., 2018
OsZFP350	C2H2	LOC_Os05g20930	5	Positively regulating primary root length, the number of adventitious and lateral roots, as well as heat, salinity and drought tolerance	Kang et al., 2019
OsZFP	C2H2	LOC_Os01g65080	1	Positively regulating the resistance to the southern rice black-streaked dwarf virus	Li, J. et al., 2017
PINE1	C2H2	LOC_Os12g42250	12	Negatively regulating internode elongation	Agarwal et al., 2007; Gómez-Ariza et al., 2019
ZFP179	C2H2	LOC_Os01g62190	1	Positively regulating salt tolerance	Sun et al., 2010
ZFP36/Bsr-d1	C2H2	LOC_Os03g32230	3	Negatively regulating seed germination under ABA treatment and blast resistance	Li, W. et al., 2017; Huang, L. et al., 2018
OsZFP	C2HC	LOC_Os01g0252900	1	Positively regulating lateral root number	Cui et al., 2017
OsDOF24	C2C2	LOC_Os08g38220	8	Negatively regulating leaf senescence, plant height, panicle length, spikelet fertility and grain numbers	Shim et al., 2019
C3H12	CCCH	LOC_Os01g68860	1	Positively regulating bacterial blight disease resistance	Deng et al., 2012
DCM1	CCCH	LOC_Os06g43120	6	Positively regulating male fertility	Zhang, C. et al., 2018
OsC3H10	CCCH	LOC_Os01g53650	1	Positively regulating drought tolerance	Seong et al., 2020
OsDOS	CCCH	LOC_Os01g09620	1	Positively regulating leaf senescence	Kong et al., 2006
OsTZF1	CCCH	LOC_Os05g10670	5	Negatively regulating seed germination, leaf senescence and photomorphogenesis of seedling	Jan et al., 2013
OsTZF5	CCCH	LOC_Os05g03760	5	Positively regulating drought resistance and grain yield	Selvaraj et al., 2020
OsRZFP34	C3H2C3	LOC_Os01g52110	1	Positively regulating stomata opening and leaf cooling.	Hsu et al., 2014
ZFP185	A20/AN1	LOC_Os02g10200	2	Negatively regulating plant height, leaf size, and panicle and internode length	Zhang, Y. et al., 2016
SNFL1	GATA	LOC_Os05g50270	5	Negatively regulating flowering time, grain size and flag leaf size	He et al., 2018
OsCOIN	RING-type	LOC_Os01g01420	1	Positively regulating tolerance to cold, salt, and drought	Liu et al., 2007
OsCESA7	RING-type	LOC_Os10g32980	10	Negatively regulating culm strengthen, plant height and fertility	Wang et al., 2016
OsCW-ZF7	CW-type	LOC_Os07g47360	7	Positively regulating awn length	Zhang et al., 2017
OsDHHC1	DHHC	LOC_Os02g57370	2	Positively regulating tiller numbers and tiller angle	Zhou et al., 2017
OsLOL2	LSD1-like	LOC_Os01g42710	1	Negatively regulating plant height but positively regulating bacterial blight resistance	Xu and He, 2007
ZFP182	TFIIIA	LOC_Os03g60560	3	Positively regulating salt and cold tolerance	Huang et al., 2007, 2012

* *Only found in the RAP database (https://rapdb.dna.affrc.go.jp/viewer/gbrowse_details/irgsp1?name=Os11g0340477)*.

## Impact on agronomic traits

Except those mentioned above, the regulation of other zinc finger genes that are involved in phytohormones underlying agronomic traits is still under-explored. For example, *O. sativa SHORT INTERNODES1* (*OsSHI1*), a plant-specific transcription factor containing the conserved zinc-finger DNA-binding domain, competes with IDEAL PLANT ARCHITECTURE1 (IPA1) to activate the expressions of *O. sativa TEOSINTE BRANCHED1* (*OsTB1*) and *O. sativa DENSE AND ERECT PANICLE1* (*OsDEP1*), resulting in the change of tiller number and panicle size (Duan et al., 2019). However, it is unclear whether *OsSHI1* mediated such regulation is related to phytohormone. A DHHC-type zinc finger gene, *OsDHHC1*, regulates plant architecture by altering rice tillering; overexpression of *OsDHHC1* increased 40% of tiller numbers and 10% of grain yield (Zhou et al., 2017). Taking into account the effect of *OsCKX2* on tiller number (Yeh et al., 2015), it raises a possibility that *OsDHHC1* may be involved in the CK pathway to modulate the tiller development.

Given that the cell morphogenesis eventually alters rice agronomic traits, identification of corresponding zinc finger proteins responsible for the cell division, differentiation, and proliferation would extend our knowledge about their roles in agronomic traits. DCM1, a CCCH zinc finger protein, has been shown to play an essential role in pollen development. Knockout of *DCM1* resulted in abnormal male meiotic cytokinesis, eventually leading to the disordered spindle orientation during meiosis and the formation of pollen grains (nearly sterile). Further analyses proposed that DCM1 might be involved in the mRNA processing or elimination by interacting with OsPABN1 and OsPABN2 to regulate callose metabolism during meiosis (Zhang, C. et al., 2018). Three cysteine-tryptophan (CW) domains containing zinc finger proteins have been reported to be involved in histone recognition, namely, OsCW-ZF3, OsCW-ZF5, and OsCW-ZF7. Among them, only knockout of *OsCW-ZF7* was examined to cause defective development of awns (Zhang et al., 2017). Investigation on the interaction between OsCW-ZF7 with histone modification and TFIID20 suggested that TFIID20-OsCW-ZFs-H3K4me module may regulate the awn development by targeting certain awn-related genes (such as *An-6/*7*/8*/*9*/*10*) in the awn primordium (Chen et al., 2018). Another zinc finger protein, NSG1, may also employ histone modification to regulate cell development. It was shown that NSG1 can recruit the corepressor TOPLESS-RELATED PROTEIN to directly bind to the promoter of *LHS1* and then inhibit the *LHS1* expression by downregulating histone acetylation levels of the chromatin, resulting in the morphological alternation of lateral organ identities in the spikelet development (Zhuang et al., 2020). A GATA zinc finger domain-containing protein, *SNFL1*, regulates flag leaf size by modulating the number of small vascular bundles rather than the number of epidermal cells (He et al., 2018). Similarly, *NECK LEAF1* (*NL1*), the allelic gene of SNFL1, was also involved in the regulation of organ size, such as leaf and panicles. However, the downstream regulators are still unknown. In addition, a ZOS4-06-C2H2 zinc-finger gene, *LRG1*, regulates spikelet development and influences grain size (Xu et al., 2020). In conclusion, zinc finger proteins comprehensively participate in the regulation of various agronomic traits. The remained issues are the identification of their targets, since the engineering of a single zinc finger would likely cause unknown side effects on other traits except the deserved ones.

### Response to abiotic stresses

**Figure 4:**
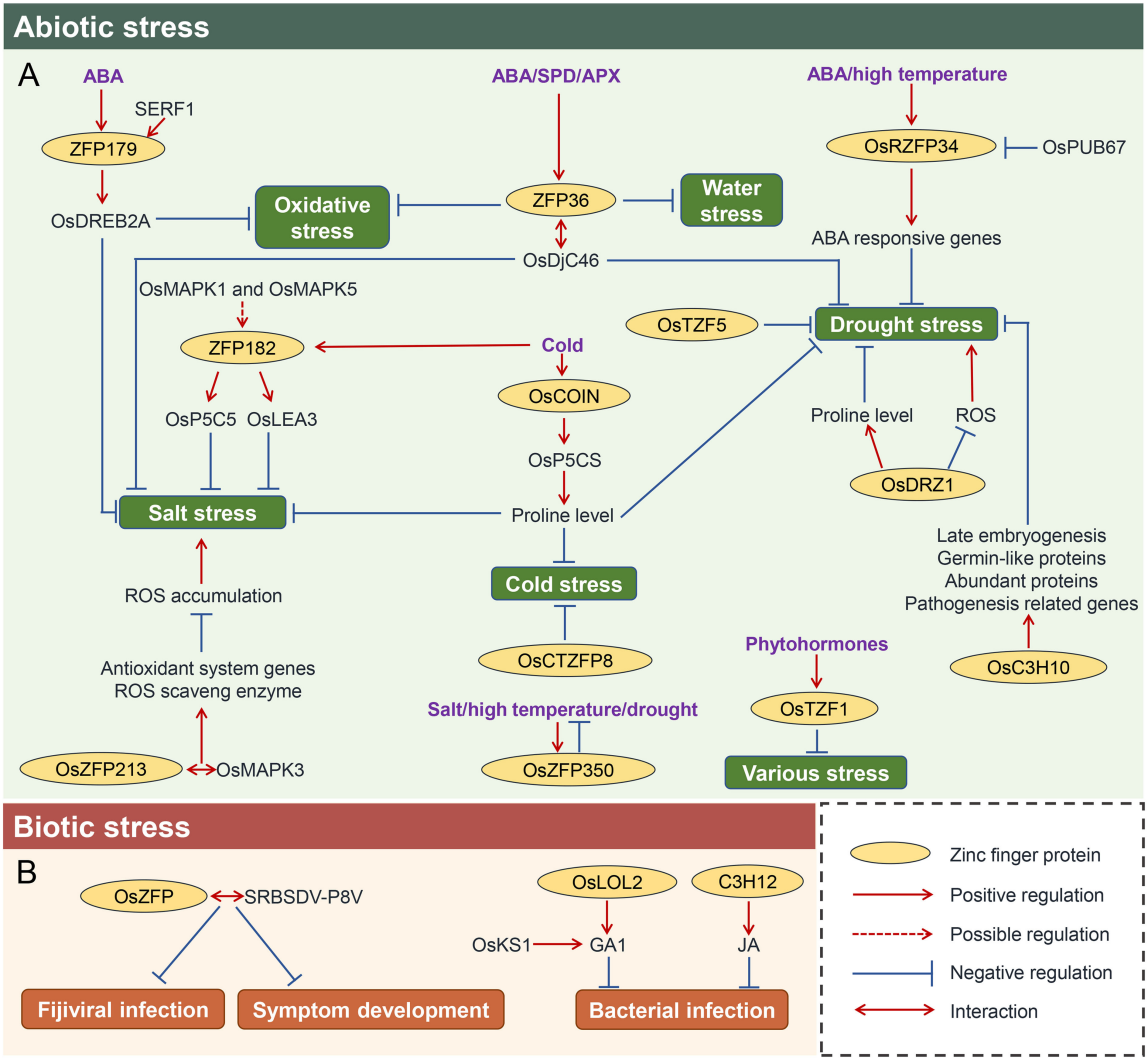
Regulations of various stresses response by zinc finger proteins in rice. The regulatory network of zinc finger protein in response to abiotic stress **(A)** and biotic stress **(B)**. Positive and negative regulations of various stresses response mediated by different zinc finger proteins are indicated by solid lines, and the proposed regulation of zinc finger proteins on downstream targets is indicated by the dashed line. The proteins with cycle are the zinc finger proteins. Double arrow represents that the two proteins interact with each other. ABA, abscisic acid; SPD, spermidine; APX, ascorbate peroxidase; JA, jasmonic acid; GA, gibberellins; SERF1, salt-responsive ERF1; OsDREB2A, dehydration responsive element binding protein; OsDjC46, heat shock protein; OsP5CS, pyrroline-5-carboxylate synthetase; OsLEA3, Late embryogenesis abundant protein gene; OsPUB67, U-box E3 ubiquitin ligase; OsTZF5, CCCH-tandem zinc finger protein 5; OsDRZ1, drought-responsive zinc finger protein 1; OsCOIN, cold-inducible zinc finger gene; OsP5CS, pyrroline-5-carboxylate synthetase; SRBSDV-P8V, black-streaked dwarf virus protein P8; OsKS1, GA biosynthetic gene.

Zinc finger proteins have been proved to regulate the responses to various abiotic stresses (Figure 4A). For example, overexpression of *ZFP179* increased the tolerance to salt and oxidative stress, enhanced the reactive oxygen species (ROS)-scavenging ability, exhibited hypersensitive to ABA, and induced the expressions of stress-related genes, such as *OsDREB2A*, suggesting *ZFP179*-mediated salt tolerance was involved both in the ABA-dependent and ABA-independent signaling pathways (Sun et al., 2010). Recently, salt-responsive ERF1 (SERF1) has been evident to bind to the promoter of *ZFP179 in vitro* and *in vivo*, and thus, overexpression of *SERF1* confers high tolerance to salt stress (Schmidt et al., 2013). *OsRZFP34* is also induced by ABA treatment, as well as high temperature, functioning in the control of stomata opening. Further analyses demonstrated that *OsRZFP34* mediated drought response by regulating the Ca^2+^ sensing, K^+^ regulator, and ABA-responsive genes (Sun et al., 2010). Subsequently, OsPUB67, a U-box E3 ubiquitin ligase, is proposed to target OsRZFP34 for degradation (Qin et al., 2020), thereby modulating the drought tolerance. Another ABA-responsive zinc finger gene *ZFP36* was also characterized in rice. Upregulation of *ZFP36* improves the tolerance to water stress and oxidative stress through induction by ABA, superoxide dismutase (SOD), and ascorbate peroxidase (APX) (Zhang et al., 2014). Later, the direct target of ZFP36 is *OsAPX1* (Huang, L. et al., 2018). Furthermore, a late embryogenesis abundant protein OsLEA5 was proved to interact with ZFP36 to co-regulate ABA-inhibited seed germination (Huang, L. et al., 2018). In addition, a heat shock protein OsDjC46 was also identified to bind ZFP36, and it positively regulates salinity and drought tolerance through the ABA pathway in rice (Huang, L. et al., 2018), suggesting ZFP36-OsDjC46 complex may function in fine-tuning the dynamic of ABA-dependent antioxidant defense. On the contrary, an *Oryza sativa* cold-inducible zinc finger gene, *OsCOIN*, can strongly be induced by low temperature. Overexpression of *OsCOIN* enhanced rice seedlings tolerance to cold, salt, and drought, accompanied by an upregulation of *pyrroline-5-carboxylate synthetase* (*OsP5CS*) and an increase in cellular proline level (Liu et al., 2007). Since *OsCOIN* is expressed in all the rice tissues (Liu et al., 2007), it is reasonable that it renders rice the low-temperature resistance at most developmental stages, which is supposed to be validated by a recent study that *OsCOIN* is also activated in response to the low temperature during the reproductive stage of rice (Guo et al., 2022). It is worthy to mention that as transcription factors, zinc finger proteins may modulate the adaptation to cold by enhancing or maintaining the growth and development of the organs. For example, overexpressing *OsCTZFP8* rendered cold tolerance with higher pollen fertilities, as well as increased seed setting and higher yield under cold treatments (Jin et al., 2018). *ZFP182* is another cold-induced zinc finger protein. Overexpression of *ZFP182* promoted rice salt tolerance in rice (Huang et al., 2007). Further analyses illustrated that *ZFP182* triggers the accumulation of compatible osmolytes, such as free proline and soluble sugars, by activating the expression of *OsP5CS* and *OsLEA3* (late embryogenesis abundant protein gene) upon stress (Huang et al., 2012). Although a ubiquitin fused to ribosomal protein L40 (ZIURP1) was found to interact with ZFP182 (Huang et al., 2012), the underlying biological significance of this interaction is poorly understood yet. However, the OsMPK1 and OsMPK5 were proved to be required for the induction of *ZFP182* in ABA-mediated antioxidant defense, while ZFP182 did not regulate the *OsMPK1* and *OsMPK5* (Zhang et al., 2012), suggesting that the *ZFP182* may act downstream of the two kinases under stress condition. Additionally, it has been shown that a zinc finger protein gene *OsZFP213* positively regulated salt tolerance in rice: overexpression of *OsZFP213* displayed enhanced salt tolerance, probably due to the upregulation of antioxidant system genes, higher catalytic activity of ROS scavenging enzymes, and lower level of ROS accumulation. OsZFP213 can also interact with a kinase, OsMAPK3, which improves the transactivation activity of *OsZFP213* to regulate salt tolerance (Zhang, Z. et al., 2018).

Accumulating studies unraveled the potential of zinc finger proteins in the drought response. For example, *OsC3H10* is involved in the regulation of rice drought tolerance by modulating the expression of stress-related genes, which included *LATE EMBRYOGENESIS ABUNDANT PROTEINs, PATHOGENESIS RELATED GENEs* and *GERMIN-LIKE PROTEINs* (Xu et al., 2020). Notably, a *drought-responsive zinc finger protein 1* (*OsDRZ1*) has been reported to play a critical role in modulating drought-responsive gene expression: overexpression of *OsDRZ1* increased seedling drought tolerance and caused accumulation of more free proline but reduced ROS, while knockdown of *OsDRZ1* led to the opposite trend (Yuan et al., 2018). Interestingly, this gene also plays a role in regulating plant architecture, implying it may orchestrate the trade-off between growth and stress.

In fact, zinc finger genes were broadly involved in multiple biological processes. For example, CCCH-type zinc finger gene, *OsTZF1*, can be induced by drought, high-salt stress, hydrogen peroxide, ABA, methylJA, and salicylic acid (SA), as well as red light (R) and far-red light (FR). Overexpression of *OsTZF1* exhibited delayed seed germination, growth retardation at the seedling stage, and delayed leaf senescence and showed improved tolerance to high-salt and drought stresses (Jan et al., 2013). Using the RNA-binding assays, it was shown that the U-rich regions in the 3'-untranslated region of messenger RNAs could bind with OsTZF1 (Jan et al., 2013). Taken together, it could be proposed that OsTZF1 may be activated by various phytohormones and then directly bind to the downstream stress-responsive genes to modulate their expressions in response to various stresses. Similar case was also identified in another zinc finger gene, *OsZFP350*, which was upregulated by drought, salt, and high temperature. Overexpression of *OsZFP350* increased primary root length and the number of adventitious and lateral roots, as well as increased the germination rate of seeds under abiotic stress, and attenuated the heat, salinity, and drought stress during root development (Kang et al., 2019). Similarly, the *Oryza sativa CCCH-tandem zinc finger protein 5* (*OsTZF5*) also confers drought resistance and increases grain yield: introducing *OsTZF5* in two commercial upland cultivars shows increased grain yield under confined field drought environments (Selvaraj et al., 2020), further suggesting that zinc finger genes play vital roles in maintaining the balance between stress and development in plants. Taken all above together, we proposed that zinc finger proteins are potential targets for simultaneously improving rice stress tolerance and yield.

### Response to biotic stresses

Increasing evidence has been implicated that zinc finger proteins also participate in biotic response in rice (Figure 4B). For example, a rice C2H2 zinc finger protein OsZFP was found to interact with southern rice black-streaked dwarf virus (SRBSDV) protein P8, a minor core protein of SRBSDV, which may play an important role in *fijiviral* infection and symptom development (Li, J. et al., 2017), implying that P8 may execute the infection by interfering with the host *OsZFP*. LSD1-related proteins in *Arabidopsis* are well known as remarkable disease-defensed zinc finger proteins. Similarly, its ortholog in rice, termed *OsLOL2*, is also involved in disease response. Overexpression of *OsLOL2* exhibited more resistance to rice bacterial blight than wild type, while decreased expression of *OsLOL2* had similar or even sensitivity to bacterial blight. Interestingly, knockdown of *OsLOL2* also resulted in dwarf phenotypes and decreased endogenous bioactive GA1 content, which can be restored by the application of GA or expression of GA biosynthetic gene *OsKS1* (Xu and He, 2007). However, it is still unclear how the GA interplays with *OsLOL2* in the culm development and whether their crosstalk is involved in the disease defense. A CCCH-type zinc finger protein, C3H12, regulates the rice-*Xanthomonas oryzae* pv. *oryzae* (*Xoo*) interaction: overexpression of *C3H12* partially enhanced resistance to *Xoo*, while knockout or suppression of *C3H12* resulted in partially increased susceptibility to *Xoo*. Since the JA levels and signaling genes were altered in *c3h12* mutants (Deng et al., 2012), it was assumed that *C3H12* may integrate into the JA pathway to induce the defense of rice.

## Concluding remarks

Breeding stress-tolerant rice with better quality and higher yield by transgenic technologies is one of the most promising approaches to combat against multiple environmental stresses including drought, salt, and extreme temperatures. The zinc finger transcription factor genes are the candidates for genetic modification, as shown in many recent reports. For example, manipulation of *ZFP182* or *OsCOIN* confers multiple stress resistances including salt, cold, and drought (Huang et al., 2007, 2012; Liu et al., 2007). Overexpression of *OsZFP350* increased the germination rate of seeds under abiotic stress and increased the resistance to heat, salinity, and drought stress during root development (Kang et al., 2019). Two genes, *OsTZF1* and *DST*, can be used to increase both salt and drought resistance (Huang et al., 2009; Jan et al., 2013). Overexpression of ZFP179 and OsZFP213 enhanced salt tolerance (Sun et al., 2010; Xu et al., 2020). *OsDRZ1, OsMSR15*, and *OsTZF5* also exhibit potential in improving drought resistance in rice (Zhang, X. et al., 2016; Yuan et al., 2018; Selvaraj et al., 2020). Notably, some zinc finger genes play antagonistic roles between biotic and abiotic stress tolerances, and thus, overexpression of these specific zinc finger genes may alter the sensitivity to both of them. For example, overexpression of a rice A20/AN1-type zinc finger gene *ZFP177* in tobacco conferred tolerance to both low- and high-temperature stresses, but increased sensitivity to salt and drought stresses (Huang et al., 2008). Therefore, ahead of extensive utilization in rice selected breeding production, these zinc finger protein genes must be tested strictly upon various biotic and abiotic stresses and then examined in the field conditions for certain years. On the contrary, because the responses of rice zinc finger proteins to stress conditions may be varied in different genetic backgrounds, it is also uncertain whether these zinc finger genes still possess the same effect capability on improving stress tolerance when they were employed in another genetic background or genotype.

Zinc finger transcription factors can be used to increase the tolerance to stresses and yield. Overexpressing *OsCTZFP8* exhibited cold-tolerant phenotypes with higher pollen fertility, increased seed setting, and higher yield under cold treatments (Jin et al., 2018). Overexpression of *OsDHHC1* increased by 40% of tiller numbers and 10% of grain yield (Zhou et al., 2017). These all indicated the potential utilization of zinc finger protein genes. It is quite interesting if and how many documented zinc finger genes are differentiated among various rice accessions. Using the database of re-sequencing of rice, we can identify their haplotypes and then evaluate their potential contributions to certain traits by integrative analyses of genome-wide association selection. As a consequence, the superior alleles would be the prime target or even directly selected for the genetic improvement of stress tolerance in rice breeding. Of the 2,408 transcription factor genes identified in *japonica* rice, there are a total of 189 C2H2 zinc finger protein genes and 74 C3H zinc finger protein genes, most of which have not been studied clearly. It is crucial to understand the regulatory mechanisms of these untested zinc finger genes before evaluating their potential in production.

## Author contributions

YH, SY, and QY conceived the idea and led the writing of the manuscript. LD, MW, and QY helped in revising the manuscript and prepared the figures. YH, LD, SY, and QY reviewed, edited, and improved the manuscript. All authors contributed to the article and approved the submitted version.

## Funding

This study was supported by the National Natural Science Foundation of China (31971920) and the Guangdong Province Key Laboratory of Plant Molecular Breeding (GPKLPMB202204).

## Conflict of interest

The authors declare that the research was conducted in the absence of any commercial or financial relationships that could be construed as a potential conflict of interest.

## Publisher's note

All claims expressed in this article are solely those of the authors and do not necessarily represent those of their affiliated organizations, or those of the publisher, the editors and the reviewers. Any product that may be evaluated in this article, or claim that may be made by its manufacturer, is not guaranteed or endorsed by the publisher.
